# Unhealthy Dietary Habits and Sedentary Behaviours Among Adolescents and Young Adults in Rome (Italy) Participating in the ALIMA Study: Growing Risks for Health

**DOI:** 10.3390/healthcare13090980

**Published:** 2025-04-23

**Authors:** Federica Intorre, Maria Stella Foddai, Eugenia Venneria

**Affiliations:** Council for Agricultural Research and Economics—Research Centre for Food and Nutrition (CREA—Food and Nutrition), Via Ardeatina 546, 00178 Rome, Italy

**Keywords:** adolescence, lifestyle, unhealthy dietary habits, sedentary behaviours

## Abstract

**Background/Objectives**: This paper aims to assess the unhealthy dietary habits and sedentary behaviours among adolescents and young adults simultaneously. **Methods**: The sample consists of 682 participants aged 10–24 years old (58.9% males and 68.2% aged 15–19 years old), recruited for the ALIMA (ALImentazione Multiculturale negli Adolescenti) study in both secondary schools and youth aggregation centres, located in Rome. The study utilizes structured questionnaires to gather data on anthropometric measurements, eating habits, alcohol consumption, physical activity, and sedentary behaviours. Data are analyzed by using the Pearson chi-squared test. **Results**: Excessive screen time (>3 h/day) and inadequate dietary habits (insufficient consumption of fruit, vegetables, and legumes, skipping breakfast, and a high consumption of sugar-sweetened beverages and alcohol) are observed in the total sample, but mainly in older adolescents aged 15–19 years old. The adherence to the MD, measured via the KIDMED index, is significantly different among the three age groups (*p* = 0.001); 47.8% of the sample has a medium adherence, while among those with a low adherence, the highest percentage is represented by older adolescents (39.8%); those with foreign-born parents have healthier diets, whereas lower parental education correlates with unhealthy behaviours. **Conclusions**: These results highlight the need for targeted interventions tailored to adolescents and young adults to encourage healthier lifestyle choices.

## 1. Introduction

Young people aged 10–24 constitute 24% of the world’s population [1]. According to the Lancet Commission on Adolescent Health and Wellbeing [2], this group includes younger adolescents (10–14 years old), older adolescents (15–19 years old), and young adults (20–24 years old) [3]. Adolescence is a transitional phase from childhood to adulthood, and it is a critical period in an individual’s physical and emotional development; this transition, corresponding to an increasing independence, takes place within a complex framework of family, peer group, community, school, and sociocultural influences and also involves lifestyle and dietary habits. Adolescents are at a crucial stage of development where correct eating habits and adequate levels of physical activity can significantly impact their health and well-being in later life too. Thus, establishing healthy eating habits during adolescence can have lasting effects into adulthood [3,4,5].

Although the family continues to be an important point of reference [6,7,8], adolescents’ growing independence in food choices is often seen in a negative light: they are increasingly engaging in unhealthy eating habits, such as eating away from home and frequently consuming energy-dense, processed “fashionable“ foods [9,10].

Anyway, the influence of parents’ educational level on adolescents’ eating habits and lifestyles is still significant and multifaceted. Scientific reports indicate that higher parental education correlates with healthier dietary behaviours among adolescents. This relationship is mediated by various factors, including the home food environment, parental modelling, and socio-economic status [11,12].

In Italy, recent studies indicate a concerning trend: many Italian adolescents are increasingly deviating from traditional Mediterranean dietary practices. This shift is attributed to several factors, including the rising popularity of junk food (fast food and processed snacks) and sugar-sweetened beverages, which include soft drinks as well as energy drinks and fruit drinks [13]. Moreover, it has been observed that there is a widespread habit of skipping breakfast and a reduction in the consumption of fruit, vegetables, and legumes [14]. Additionally, the influence of social media and peer pressure often leads to unhealthy eating behaviours among adolescents that prioritize convenience over nutritional value. As the Mediterranean Diet (MD) offers numerous health benefits for adolescents, promoting not only physical well-being but also mental health, encouraging young people to adopt this dietary pattern can foster healthier lifestyles that may persist into adulthood, ultimately reducing the risk of chronic diseases [4].

Regular breakfast consumption is a significant indicator of healthy eating habits, linked to various positive health outcomes. Studies have shown that individuals who regularly eat breakfast tend to have a healthier Body Mass Index (BMI), improved mental health, and higher intakes of essential micronutrients. Furthermore, breakfast eaters often exhibit better cognitive performance and overall diet quality, which is crucial for adolescents who may face health issues due to poor eating habits, such as overweight, obesity, or underweight conditions. Adolescents with poor eating habits or whose weight is outside of the normal range (underweight or overweight and obesity) can experience various health problems and poorer school achievement [15,16,17,18,19].

Among sugar-sweetened beverages, energy drinks are the most popular among young people [20,21,22]. Energy drinks are non-alcoholic beverages that contain high levels of caffeine and sugar, along with other stimulants, which may interact with caffeine. A high caffeine intake can result in caffeine intoxication and dependence, while a high sugar intake is linked to weight gain, obesity, type 2 diabetes, and dental erosion [16]. Additionally, it has been proposed that energy drink consumption could be a risk factor for alcohol use among adolescents. One theory suggests that the high levels of caffeine and taurine in energy drinks may impact the brain’s reward system, altering its response to alcohol. Another explanation is that combining alcohol with energy drinks counteracts the sedative effects of alcohol, enhancing its stimulating effects. Importantly, this suggests that the connection between energy drinks and alcohol use may be influenced by underlying factors such as inadequate parenting or peer pressure [23]. As adolescents seek independence, it triggers them to experiment with alcohol. Peer pressure plays a significant role in this behaviour, as many youths feel compelled to drink to fit in or gain acceptance within their social circles [24,25,26]. Adolescent alcohol consumption is a significant public health concern, with various studies highlighting its prevalence and associated health risks, impacting both physical and mental health [27]. The early initiation of drinking is linked to a higher risk of developing alcohol use disorders later in life. Moreover, those who begin drinking before age 15 are four times more likely to develop a dependence compared to those who start at age 21 or older [28].

In addition to diet, physical activity is a modifiable risk factor influencing health conditions. Physical activity is crucial for adolescents for several reasons: participation in recreational and sporting physical activities represents an opportunity for young people to improve themselves, overcome their limits, and have fun. It is widely demonstrated that the most evident benefits that a young person derives from practising regular physical activity are manifested not only in organic development but also in social behaviour and autonomy [3].

The World Health Organization (WHO) has updated its physical activity guidelines for children and adolescents. One key change was the recommendation for moderate-to-vigorous physical activity, which shifted from 60 min daily in 2010 to an average of 60 min per day in 2020 [29,30]. Children and adolescents aged 5–17 are also advised to engage in vigorous physical activity at least three times a week and reduce sedentary behaviour. For adults (18–64 years old), it is recommended to engage in at least 150–300 min of moderate-intensity aerobic activity, or at least 75–150 min of vigorous-intensity aerobic activity, or an equivalent combination of both types of activity throughout the week, to achieve significant health benefits [30].

However, one notable trend is the reduction in physical activity, which often becomes more pronounced as individuals transition from early to late adolescence, replaced by more sedentary activities. The sedentary lifestyle of young people is related to the rapid expansion of digital technology: the electronic revolution seems to have changed the movement patterns of adolescents, encouraging them to sit more, walk less, and be less active in general [31]. The evidence suggests that an increased time spent on sedentary activities is linked to negative health outcomes [30]. However, there was not enough evidence to establish a specific threshold (or “cut-off”) for sedentary or recreational screen time [30]. Establishing a habit of regular physical activity during adolescence can lead to continued engagement in healthy behaviours into adulthood, resulting in a lower risk of chronic diseases and an overall healthier lifestyle [3].

Hence, as a comprehensive understanding of adolescent-onset risk behaviours is essential to prevent the onset of chronic diseases [6,7,8], the aim of this paper is to evaluate, simultaneously, unhealthy dietary habits and sedentary behaviours among adolescents and young adults (10–24 years old). This research stems from the need to further investigate the critical issues pertinent to this broad age group, given the limited availability of studies in Italy that simultaneously address these interconnected aspects.

## 2. Materials and Methods

### 2.1. Participants and the Study Design

Volunteers were recruited in both secondary schools and youth aggregation centres located within seven different municipalities, strategically distributed across the urban territory of the city of Rome, from October 2020 to June 2024 and included 682 adolescents and young adults aged 10–24 years old (total sample: mean age 16.0 ± 2.4 years old; males: mean age 16.1 ± 2.6 years old; females mean age 15.8 ± 2.2 years old). Both secondary schools and youth aggregation centres, where the volunteers were recruited, are either partnered with CREA—Food and Nutrition or voluntarily enrolled in Roma Capitale’s “Mappa della Città Educante 23–24” project for the implementation of the ALIMA (ALImentazione Multiculturale negli Adolescenti) study, whose aim is to evaluate if and to what extent the encounter between different cultures and geographic backgrounds, in the context of globalization present in modern society, has influenced the lifestyle and eating habits of adolescents. After obtaining informed consent, qualified interviewers administered questionnaires in a “face to face” assisted modality, on one occasion. They were designed to obtain information about lifestyle, eating habits, food consumption, adherence to the MD, physical activity, and sedentary behaviour; all the data were anonymously collected to maintain and protect confidentiality and handled according to the European General Data Protection Regulation—GDPR 679/2016. As the assessment did not involve invasive procedures or induce changes in dietary patterns, the approval by the Ethics Committee was not necessary.

### 2.2. Lifestyle and Eating Habits

Information about lifestyle and eating habits was collected through a questionnaire, described in detail elsewhere [32,33,34]. Briefly, it consists of a package of questions specifically designed to obtain different information about demographic factors and social aspects (parents’ geographical background and educational level), eating habits (breakfast, water, energy drinks, soft drinks, and alcohol consumption).

### 2.3. Anthropometric Data

Self-reported weight and height were used for calculating the BMI (kg/m^2^), and consequently for classifying volunteers into underweight (<18.5 kg/m^2^), normal weight (18.5–24.9 kg/m^2^), overweight (25.0–29.9 kg/m^2^), and obese (≥30.0 kg/m^2^) [35].

### 2.4. Adherence to the MD

The adherence to the MD was calculated by reference to the KIDMED index [36,37]. The KIDMED questionnaire consists of 16 dichotomous (positive/negative) items, where the questions denoting a negative connotation to the MD are scored with −1, and the questions denoting a positive connotation are scored with +1. The score obtained summing the single answers allow the classification of the adherence to the MD into three levels: low (≤3), medium (4–7), and high (≥8).

### 2.5. Physical Activity and Daily Screen Time

Physical activity was assessed via the Godin–Shephard Leisure-Time Physical Activity Questionnaire, composed of 3 questions about the number of times spent in physical activity with 3 different intensities (strenuous, moderate, and light) in a typical 7-day period. The total physical activity is expressed in arbitrary units, according to the formula: Weekly score = (9 × Strenuous physical activity) + (5 × Moderate physical activity) + (3 × Light physical activity). The total score allows the subjects to be classified into three levels: total score ≥ 24, active; total score 14–23, moderately active; total score < 14, sedentary [38,39]. An additional questionnaire was administered, to assess the participants’ daily screen time during the weekdays (Monday to Friday) and separately during the weekend.

### 2.6. Statistical Analysis

MedCalc Software version 23.1.3 for Windows was used to perform all the statistical analyses. The analyses were conducted using the maximum number of subjects for whom data were available (*N* = 682): this number decreased in the analysis considering the BMI categories (*N* = 423) and parental educational level (*N* = 531). Categorical variables were reported as percentages, and the associations among them were tested by using the Pearson chi-squared test, establishing the significance at *p* < 0.05. A multiple logistic regression model was employed to evaluate the association, reported as an adjusted odds ratio (AOR) and 95% confidence intervals (CI), among dietary and lifestyle habits and demographic and anthropometric characteristics, as gender, age, and parental geographical background. Both the BMI and parents’ education level were excluded from the regression due to missing data, but subsamples with complete data were created to perform the statistical analysis.

## 3. Results

The data reported in Table 1 summarize the various demographic and anthropometric characteristics of the study sample, consisting of 682 volunteers, across different age groups, i.e., younger adolescents (10–14 years old), older adolescents (15–19 years old), and young adults (20–24 years old). Most of the sample (68.2%) was aged 15–19, as they were mainly recruited in secondary schools, and males (58.9%). Regarding anthropometry, it is noteworthy that about 38.0% of the volunteers did not know or remember their anthropometric measures, and for them it was impossible to determine their BMI. For the other volunteers, their BMI was mainly in the normal range (41.8%), while the remaining sample was distributed between underweight (10.6%) and overweight/obesity (8.2% and 1.5%), which are both risk factors for health. Moreover, most of the underweight participants were 10–14 (17.6%), while those who were overweight and obese were mainly young adults (12.1% and 1.7%, respectively) (*p* = 0.001). Most of the adolescents (60.6%) had parents who were both Italian, whereas 39.4% had at least one parent hailing from another country. Regarding the parental educational level, 4.4% of the adolescents lacked external care influencing their lifestyle and 22.1% did not know this datum; however, most of the parents (27.1%) had a secondary education.

In Table 2, healthy dietary habits are reported by age group. The data presented indicate significant differences in dietary habits among age groups, specifically concerning daily breakfast consumption, the adherence to the MD, and the intake of vegetables, legumes, and water. Only 46.2% of the volunteers had daily breakfast. Statistical analysis shows significant differences among the three age groups (*p* = 0.016): adolescents aged 15–19 years old were the ones with the worst behaviour, with 57.2% skipping breakfast daily. Of the total sample, 15.1% never have breakfast. The results demonstrate that the adherence to the MD, measured via the KIDMED index, is significantly different among the three age groups (*p* = 0.001). Of the sample, 47.8% had a medium adherence, while among those with a low adherence, the highest percentage is represented by the older adolescents (39.8%). Of the sample, 45.3% and 46.8% do not eat fruit or vegetables, respectively, at least once a day. Older adolescents are those with the worst behaviour, even if there are no statistically significant differences among the age groups for fruit consumption, while the *p*-value is 0.05 for vegetable consumption. Of the sample, 57.6% do not consume legumes, especially the older adolescents (*p* = 0.001). Regarding water consumption, 14.7% of the sample did not know the quantity of water they drink, and the majority of the sample (65.1%) do not reach the recommended daily quantity of water consumption (almost 1.5 L). There are statistically significant differences among the age groups (*p* = 0.001).

Table 3 presents the data on unhealthy dietary habits among the different age groups, focusing on the consumption of energy drinks, soft drinks, and fast food. The analysis highlights that, while energy drink and fast food consumption remain relatively stable across the age groups, soft drink consumption shows significant variations, particularly peaking in the older adolescents (21.3%) (*p* = 0.014). The 20–24 age group had a significantly lower daily consumption rate (5.2%) but showed a higher percentage of weekly consumption (43.1%). Regarding energy drinks, young adults do not consume them daily, and the highest percentage was in the 10–14 age group (6.3%). Even though the percentages of those who consume them daily are low, 20.2% of the total sample reports consuming energy drinks weekly. The data indicate that younger and older adolescents (aged 10–14 and 15–19 years old) are more likely to consume energy drinks and soft drinks regularly compared to young adults (20–24 years old). Concerning fast food consumption, more than one in four of the volunteers (27.1%) goes to a fast food restaurant at least once a week. Although the differences are not statistically significant, older adolescents fare the worst (29.0% of them go to fast food restaurants at least once a week).

Table 4 reports the prevalence of alcohol consumption across different age groups. It is important to underline that 28.9% of younger adolescents drink alcohol. It is noteworthy that 18% of the total sample do not consume alcohol due to religious reasons. The findings indicate significant differences (*p* = 0.001) in behaviours related to alcohol consumption, and binge drinking (defined in Italy as the consumption of six or more alcoholic drinks on a single occasion at least once in the past 12 months).

The data provided in Table 5 outline the distribution of daily screen time and physical activity across the different age groups. Most of the individuals across all the age groups exceed three hours of screen time daily, with no significant differences noted. Younger adolescents are more likely to be classified as active, compared to the other age groups (*p* = 0.001).

Table 6 describes the adjusted odds ratio (AOR) and 95% confidence intervals (CIs) showing the association of dietary habits with gender, age, and parents’ geographic background, obtained from a multiple regression model. The data show a significant difference in breakfast skipping among the age groups. Specifically, individuals aged less than 15 and over 19 years old have an AOR of 0.66 (95% CI: 0.48–0.91, *p* = 0.012), showing that they are less likely to skip breakfast compared to those aged 15–19 years old. Regarding the adherence to the MD, the AOR for individuals aged less than 15 and over 19 years old is 0.54, (95% CI: 0.38–0.77, *p* = 0.001). This indicates that those in these age groups are significantly more likely to adhere to the MD compared to the reference group of 15–19 years old. Moreover, adolescents with foreign-born parents show an AOR of 0.58 (95% CI: 0.42–0.82, *p* = 0.002), suggesting that they are significantly more likely to adhere to the MD compared to those whose parents are Italian. The results for fruit consumption show that the age group <15 and >19 years old has an AOR of 0.70 (95% CI: 0.50–0.97, *p* = 0.034), meaning a statistically significant lower likelihood of not consuming fruit compared to the reference group (aged 15–19 years old). The data also reveal that individuals with foreign parents have an AOR of 0.51 (95% CI: 0.37–0.70) and a highly significant *p*-value of 0.001, suggesting that individuals from foreign backgrounds are significantly less likely to be non-consumers of fruit compared to those with Italian parents, indicating that cultural factors may play a role in dietary habits. The results presented indicate a significant association between vegetable consumption and gender and age. The AOR for females not consuming vegetables is 0.58 (95% CI: 0.43–1.80, *p* = 0.001), showing a statistically significant less likelihood to consume vegetables compared to males. Regarding age, the AOR for individuals aged less than 15 or over 19 years old compared to those aged 15–19 years old is 0.65 (95% CI: 0.47–0.91, *p* = 0.011), suggesting that older adolescents might be at a higher risk associated with no vegetable consumption than their younger and older counterparts. Significantly, the consumption of legumes among adolescents aged 15–19 years old is intake (AOR: 0.58, 95% CI: 0.41–0.80, *p* = 0.001); the data also reveal that individuals with foreign-born parents have a greater likelihood of consuming legumes (AOR: 0.54, 95% CI: 0.40–0.75, *p* = 0.001).

Table 7 describes the AOR and 95% confidence intervals (CIs) showing the association of lifestyle and other dietary habits with gender, age, and parents’ geographic background, obtained from a multiple regression model. The results presented for alcohol consumption reveal significant differences based on age and parents’ geographic background. Regarding age, the AOR is 0.47 (95% CI: 0.34–0.66) with a highly significant *p*-value (0.001). This indicates that adolescents aged <15 and >19 years old are significantly less likely to consume alcohol compared to the reference group (15–19 years old). Moreover, the AOR for individuals with foreign parents is 0.59 (95% CI: 0.43–0.80, *p* = 0.001), implying that individuals from foreign backgrounds may have lower odds of alcohol consumption compared to those with Italian parents. This datum is probably due to some foreigners (18%) not consuming alcohol due to religious reasons. The analysis of daily screen time and its implications reveals several noteworthy insights, particularly concerning age differences. Those aged <15 and >19 years old show an AOR of 0.59 (95% CI: 0.37–0.94, *p* = 0.027) for excessive screen time, suggesting that the adolescents in these groups may engage less in excessive screen time compared to other age brackets (15–19 years old). Finally, females are significantly more likely to be physically inactive compared to males, with an AOR of 1.62 (95% CI: 1.02–2.59, *p* = 0.043).

Both the BMI and parents’ education level were excluded from the regression due to missing data. However, subsamples with complete data were created, and while the BMI does not significantly affect the selected variables, it was observed that parents’ educational level affects breakfast habits as well as the consumption of vegetables, energy drinks, soft drinks, and fast food. Specifically, the findings reveal that volunteers with parents who have a low educational level are more likely to engage in unhealthy eating behaviours, compared to those with parents who have a high educational level. In particular, the results show that volunteers with parents with a low educational level have a greater likelihood of skipping breakfast (AOR 1.64, 95% CI: 1.14–2.36, *p* = 0.007), drinking energy drinks at least once a day (AOR 3.19, 95% CI: 1.51–6.72, *p* = 0.002), drinking soft drinks at least once a day (AOR 1.94, 95% CI: 1.23–3.05, *p* = 0.005), and visiting fast food restaurants at least once a week (AOR 1.82, 95% CI: 1.22–2.71, *p* = 0.003) and are less likely to consume vegetables (AOR 0.61, 95% CI: 0.43–0.88, *p* = 0.007), compared to those with a parents with a high educational level. The interactions among the different unhealthy lifestyle and dietary habits have been analyzed, but it is noteworthy that the AOR values only show the association of daily soft drink consumption with alcohol consumption, energy drink consumption, fast food intake, and physical inactivity. The observed associations may suggest overlapping behavioural patterns in dietary choices. Alcohol consumers are nearly twice as likely to consume soft drinks daily (AOR 1.94, 95% CI: 1.26–3.00) compared to those who do not drink alcohol, with a statistically significant *p*-value of 0.003. Moreover, individuals who consume energy drinks are over 12 times more likely to also consume soft drinks daily (AOR 12.46, 95% CI: 5.60–27.70, *p* = 0.001). The AOR for individuals consuming fast food at least once a week is 2.65 (95% CI: 1.70–4.11), suggesting that frequent fast food consumers are more than twice as likely to consume soft drinks daily, with a *p*-value of 0.001. This relationship may highlight the connection between convenience foods and sugary beverage choices, as both are often linked to poor dietary habits. Finally, sedentary individuals who have an AOR of 2.10 (95% CI: 1.17–3.75, *p* = 0.013) are more likely to consume soft drinks daily compared to those who are active.

## 4. Discussion

The most recent literature data on Italian adolescents that investigate health-related behaviours come from the Health Behaviour in School-aged Children (HSBC) surveillance system that monitors Italian adolescents aged 11–17 years old [40]. A shift has been observed towards poor dietary habits and incorrect lifestyles. As this age range does not coincide with our classification, it could be difficult to compare the data. Indeed, three age groups—younger adolescents (10–14 years old), older adolescents (15–19 years old), and young adults (20–24 years old) [2,3]—each of them experiencing distinct developmental milestones and challenges—have been considered in the present research, whose aim is to provide a comprehensive overview of the demographic and lifestyle characteristics of adolescents, highlighting unhealthy dietary habits and sedentary behaviours.

The results for the anthropometric status reveal significant differences across the age groups. Most of the underweight participants were 10–14, while the overweight and obese participants were mainly young adults. It is important to underline that self-reported weight and height might not correspond to the true physiological values: self-reported height tends to be slightly overestimated, and weight underestimated, resulting in an underestimation of the BMI. However, the use of self-reported anthropometric measurements can be used for weight classification purposes [41]. Is it noteworthy that about 38.0% of the volunteers did not know or remember their anthropometric measurements, and for them it was impossible to determine their BMI. The high percentage of “Don’t know” responses (43.4% for younger adolescents) indicates a lack of body awareness among this group, which could be linked to developmental factors or insufficient education on health-related topics. Studies indicate that interventions aimed at increasing health literacy can significantly improve people’s knowledge about body awareness and the related health risks among adolescents [42,43]. This indicates that enhancing the education around this issue could mitigate the high percentage of “Don’t know” responses by fostering a more informed adolescent population. The lack of awareness about the BMI can result in poor dietary choices and physical inactivity, increasing the health risks [42,43].

The influence of parental educational level on the BMI is well documented in the literature, revealing that a higher parental education is generally associated with a lower BMI [44,45]. This association can be attributed to various factors, including lifestyle choices and nutritional knowledge. Numerous studies have established an inverse relationship between parental education levels and obesity rates; for instance, the children of parents with a lower educational attainment are more likely to be classified as overweight or obese [44,45]. In our study, the parents’ educational level did not affect the BMI values but significantly affected their children’s breakfast habits as well as their consumption of vegetables, energy drinks, soft drinks, and fast food; specifically, the findings reveal that the volunteers whose parents had a low educational level were more likely to engage in unhealthy eating behaviours.

The findings regarding the dietary behaviours among the volunteers correlate well with the existing literature emphasizing the impact of parental education on eating habits. Lower educational levels appear to contribute significantly to unhealthy dietary practices such as skipping meals and an increasing consumption of sugar-sweetened beverages and fast food and reduction in vegetable intake, which can lead to long-term health implications [46,47,48,49,50].

The results of the dietary habits analyzed in this study reveal significant differences in consumption patterns based on age, with older adolescents often exhibiting poorer dietary choices compared to younger adolescents and young adults. For instance, while younger adolescents and young adults are more likely to eat breakfast, older adolescents appear to skip it more frequently. In particular, while gender and parental geographic background do not show strong associations with skipping breakfast, age appears to be a significant factor. This general waning during late adolescence could be linked to lifestyle changes, increased social activities, or a change in priorities that often occurs during this developmental stage. A recent systematic review on the prevalence of breakfast skipping among children and adolescents across 33 countries highlights that 10–30% of young people skip breakfast [51]. Breakfast skipping is often attributed to factors such as time constraints, lack of enjoyment, absence of hunger, and weight management concerns. It is particularly common among females, older children, and adolescents and frequently coexists with other unhealthy habits [50,51,52]. These results align with broader discussions about the health impacts of skipping breakfast, including the increased risks of obesity, cardiovascular disease, and metabolic disorders [53]. For instance, studies indicate that those who regularly skip breakfast may face a higher likelihood of developing conditions such as hypertension and type 2 diabetes [54]. Additionally, skipping breakfast can affect cognitive function and mood, potentially leading to irritability and decreased concentration [55].

Another considered aspect in this study is the adherence to the MD, which was less among older adolescents aged 15–19 years old, reflecting a lower health awareness or lifestyle choices being influenced by education or social circles and raising concerns about the dietary quality during this critical growth period. Moreover, the higher adherence rates to the MD among individuals with foreign-born parents may point to cultural differences in dietary habits and preferences, as immigrant families might maintain traditional eating patterns that align more closely with the MD. Understanding these cultural backgrounds can inform the targeted interventions aimed at improving the dietary adherence among different age groups and cultural contexts [34,56,57]. Studies show that a significant percentage of adolescents consume diets high in processed foods while lacking in fruit, vegetables, and legumes, further exacerbating the issue of poor adherence to the MD [49,56,57,58,59].

The consumption of fruit and vegetables at a national level is very low: only 32.7% of adolescents eat fruit at least once a day every day; vegetable consumption is even lower, with only 27.8% eating vegetables daily. Moreover, 47.3% of adolescents do not eat legumes at least twice a week [40], as recommended [14]. In our study, 45.3% and 46.8% of the sample did not eat fruit or vegetables, respectively, at least once a day, and 57.6% did not eat legumes at least twice a week. Older adolescents exhibit the poorest eating habits. The overall evidence supports the conclusion that increased fruit and vegetable consumption is linked to reduced risks of various health issues [60,61,62]. Legumes are recognized for their health benefits, including reduced risks of cardiovascular disease, obesity, and improved metabolic health due to their high fibre and protein content; regular consumption is linked to better weight management and lower cholesterol levels, which are critical factors in preventing chronic diseases [63,64,65]. The findings from this study reinforce the importance of promoting their intake across different demographics, especially targeting groups identified as at risk based on their gender, age, and parental geographic background.

Contrary to recommendations, adolescents are increasingly consuming energy-dense, nutrient-poor food, including sugar-sweetened snacks and beverages. Our results show that daily soft drink consumption is significantly higher in older adolescents, aged 15–19 years old. These results also highlight the significant associations between soft drink and energy drink consumption as well as fast food intake. The literature studies indicate that higher soft drink intake correlates with increased risks of obesity and lower nutrient intake among adolescents; for instance, a global study highlighted a positive association between daily soft drink consumption and obesity prevalence among adolescents across multiple countries [66,67].

Regarding fast food consumption, the results for adolescents aged <15 and >19 years old, recruited in our study, suggest a lower consumption compared to older adolescents aged 15–19 years old, although this result is not statistically significant. While specific data on fast food consumption are not detailed for Italy, it is noted that Italian adolescents have high intakes of nutrient-poor food [13]. The consistent findings across various studies emphasize the pervasive role of fast food in modern diets, with significant implications for public health given its association with a poorer dietary quality and health outcomes [68,69]. The trend towards fast food consumption is also notable, as adolescents are more likely to snack frequently and skip meals compared to younger children [10].

The energy drink consumption among adolescents is a significant public health concern due to its high prevalence and potential health risks [20]. Studies have shown that the prevalence of energy drink consumption among Italian adolescents is about 57% [22], and over the past decade, there has been a substantial increase [21]. While university students also consume energy drinks, the prevalence seems lower compared to high school students [70,71]. For instance, one study reported that about 15.2% of university undergraduates consumed caffeinated energy drinks over a six-month period. Moreover, energy drink consumption tends to be higher among males than females across different age groups in Italy [70,71]. Our findings indicate a greater consumption among younger adolescents; however, this difference is not statistically significant. Additionally, no statistically significant gender effect was observed, and while there is a notable difference, it may not be robust enough to draw definitive conclusions. A concerning trend is the mixing of energy drinks with alcohol, which can mask the intoxication symptoms and lead to dangerous effects such as increased dehydration and alcohol dependency risks [16,71]. Approximately 49% of adolescent consumers mix their energy drinks with alcohol [22].

The results for alcohol consumption, described in this study, reveal significant differences based on age and the parental geographical background. Regarding age, adolescents aged <15 and >19 years old are significantly less likely to consume alcohol compared to the reference group of 15–19 years old. Moreover, the AOR for individuals with foreign parents implies that they may have lower odds of alcohol consumption compared to those with Italian parents, possibly due to cultural and religious reasons, as previously declared. These results highlight the critical factors influencing the alcohol consumption patterns among adolescents and young adults. The stark contrast in drinking behaviours by age and parental background suggests that targeted interventions could be beneficial, particularly focusing on younger demographics who show lower consumption rates but may be at risk as they face the transition into adulthood. In 2022, around 650,000 minors aged 11–17 consumed alcohol in Italy, with 17.5% of males and 15.5% of females reporting use. Binge drinking affected 10.5% of males and 3.7% of females over the age of 11, totalling about four million individuals. The prevalence increased during adolescence, peaking at ages 18–24 (18.9% for males, 10.8% for females), and then declined with age [72].

Among the aspects considered, physical activity and sedentary behaviour hold particular importance for adolescents [73,74]. Common sedentary activities include watching TV, playing video games, and using computers, collectively referred to as “screen time,” as well as reading. Exposure to screen media can also contribute to unhealthy eating habits, characterized by a high consumption of energy-dense foods and sugary drinks during viewing. Regarding that, the WHO strongly advises that children and adolescents limit their sedentary time, especially recreational screen time [30]. The analysis of the daily screen time, carried out in this study, and its implications reveal several noteworthy insights, particularly concerning age differences. Indeed, those aged <15 years old and >19 years old show a significantly lower odds ratio for excessive screen time, suggesting that the adolescents in these groups may engage less in excessive screen time compared to other age brackets (15–19 years old). Excessive screen time has been linked to various health issues across studies. Research indicates associations with obesity, mental health disorders such as depression and anxiety, and negative impacts on cognitive functions [75,76]. The CDC reports that over 50% of teenagers aged 12–17 engage in four or more hours of daily screen time [77], underscoring a significant public health concern.

Moreover, the results highlight the gender differences in physical inactivity. In particular, the analysis indicates that females are significantly more likely to be physically inactive compared to males. This finding aligns with broader research indicating that males generally engage in more physical activity than females [78,79]. Physical activity is a healthy behaviour that is significantly affected by the growing academic, household, and occupational responsibilities during adolescence, all of which are deeply intertwined with the social context of this age group. Despite its numerous health benefits, it is alarming that only about 20% of adolescents meet the previous recommendation of engaging in at least 60 min of moderate-to-vigorous-intensity physical activity daily. Moreover, these already concerning levels are decreasing in most countries [80]. Considering that females generally exhibit lower levels of physical activity than males worldwide, the results of this study underscore the need for both macro- and micro-level changes to effectively boost the overall physical activity among adolescents and promote greater equity in this area.

## 5. Strengths and Limitations

This study has the limitation that the reliance on self-reported data introduced potential biases and inaccuracies, impacting the reliability of the findings. Moreover, as previously mentioned, weight and height as self-reported and not measured might not correspond to the true values, even if they can be used for weight classification purposes [43]. The sample size and the geographically limited area do not allow generalizations and extrapolations; thus, the study is ongoing, involving larger populations, recruited in different municipalities. Despite these limitations, this study also has some strengths. The most important relates to the fact that the participants are part of a population group that has not been widely assessed. Moreover, questionnaires are not emailed or sent through an online system but instead are completed during face-to-face interviews. This method allows for the immediate verification of responses, minimizing incomplete or inconsistent reporting and ultimately improving the data quality.

## 6. Conclusions

This study contributes data of interest on the dietary habits and lifestyles of adolescents and young adults. Inadequate dietary habits were observed in the total sample, but mainly in older adolescents aged 15–19 years old, underscoring the necessity for targeted interventions tailored to this age group. Effective strategies could involve public health initiatives and educational programmes designed to promote healthier dietary choices and lifestyles. By integrating cultural perspectives and implementing targeted interventions, it may be possible to foster healthier eating practices during this critical developmental stage. Schools and community programmes are pivotal in educating adolescents about the benefits of healthy eating habits and lifestyles, encouraging them to make informed food choices that respect their cultural background. Moreover, the potential health risks linked to unhealthy behaviours emphasize the need for awareness and possibly regulatory measures to safeguard vulnerable populations like adolescents. Addressing this issue demands a comprehensive approach that combines education, community engagement, and family support to encourage healthier eating patterns that contribute to lifelong well-being.

## Figures and Tables

**Table 1:** Study sample characteristics by age group.

	Age Groups				
	10–14	15–19	20–24	All	*p*-Value
***N* (%)**	159 (23.3)	465 (68.2)	58 (8.5)	682	
**Gender (%)**					ns
Males	58.5	57.4	72.4	58.9	
Females	41.5	42.6	27.6	41.1	
**BMI categories (%)**					0.001
Underweight	17.6	8.8	5.2	10.6	
Normal weight	35.2	41.2	65.5	41.8	
Overweight	3.8	9.2	12.1	8.2	
Obesity	0.0	1.9	1.7	1.5	
Do not know	43.4	38.9	15.5	37.9	
**Parents’ geographic background (%)**					0.001
Both Italians	66.7	61.3	37.9	60.6	
One or both foreigners	33.3	38.7	62.1	39.4	
Parents’ educational level (%) *					0.001
None	2.5	3.4	8.6	3.7	
Primary education	14.5	22.2	6.9	19.1	
Secondary education	30.2	25.6	31.0	27.1	
Higher education	25.2	22.4	29.4	23.6	
No external cares	0.6	6.2	0.0	4.4	
Do not know	27.0	20.2	24.1	22.1	

Data are presented as percentage for categorical variables. Statistical analysis: chi-squared test; ns = not significant. * The highest educational level between the two parents or of someone in their stead.

**Table 2:** Healthy dietary habits by age group.

	Age Groups				
	10–14	15–19	20–24	All	*p*-Value
**Daily breakfast (%)**					0.016
Yes	50.9	42.8	60.3	46.2	
No	49.1	57.2	39.7	53.8	
**Adherence to the Mediterranean Diet (%)**					0.001
Low	27.7	39.8	22.4	35.5	
Medium	47.8	47.1	53.4	47.8	
High	24.5	13.1	24.1	16.7	
**Fruit at least once a day (%)**					ns
Yes	61.6	51.8	58.6	54.7	
No	38.4	48.2	41.4	45.3	
**Vegetables at least once a day (%)**					0.050
Yes	61.0	50.1	56.9	53.2	
No	39.0	49.9	43.1	46.8	
**Legumes at least twice a week (%)**					0.001
Yes	47.8	38.1	62.1	42.4	
No	52.2	61.9	37.9	57.6	
**Water at least 1.5 L/day (%)**					0.001
Yes	20.8	16.6	48.3	20.2	
No	59.7	69.5	44.8	65.1	
Do not know	19.5	14.0	6.9	14.7	

Data are presented as percentage for categorical variables. Statistical analysis: chi-squared test; ns = not significant.

**Table 3:** Unhealthy dietary habits by age group.

	Age Groups				
	10–14	15–19	20–24	All	*p*-Value
**Energy drinks at least once a day (%)**					ns
Yes	6.3	6.0	0.0	5.6	
No	93.7	94.0	100.0	94.4	
**Soft drinks at least once a day (%)**					0.014
Yes	20.1	21.3	5.2	19.6	
No	79.9	78.7	94.8	80.4	
**Fast food at least once a week (%)**					ns
Yes	23.9	29.0	20.7	27.1	
No	76.1	71.0	79.3	72.9	

Data are presented as percentage for categorical variables. Statistical analysis: chi-squared test; ns = not significant.

**Table 4:** Alcohol consumption by age group.

	Age Groups				
	10–14	15–19	20–24	All	*p*-Value
**Alcohol consumption (%)**					0.001
Yes	28.9	56.3	62.1	50.4	
No	71.1	43.7	37.9	49.6	
**Binge drinking (%)**					0.001
Yes	2.5	18.5	20.7	15.0	
No	21.4	28.4	36.2	27.3	
Do not know	5.0	9.5	5.2	8.1	
Teetotaller	71.1	43.7	37.9	49.6	

Data are presented as percentage for categorical variables. Statistical analysis: chi-squared test; ns = not significant.

**Table 5:** Daily screen time and physical activity by age group.

	Age Groups				
	10–14	15–19	20–24	All	*p*-Value
**Daily scree time (%)**					ns
<3 h	17.0	10.1	13.8	12.0	
>3 h	83.0	89.9	86.2	88.0	
**Physical activity (%)**					ns
Active	81.0	72.2	74.1	74.4	
Moderately active	9.5	15.1	13.8	13.7	
Inactive	9.5	12.7	12.1	11.9	

Data are presented as percentage for categorical variables. Statistical analysis: chi-squared test; ns = not significant.

**Table 6:** Adjusted odds ratio (AOR) and 95% confidence intervals (CIs) from logistic regression analysis showing the association of dietary habits with gender, age, and parents’ geographic background.

	Skipping Daily Breakfast		Low Adherence to the MD		No Fruit Consumption		No Vegetable Consumption		Legumes Consumption < 2 Times a Week	
	AOR (95% CI)	*p*-Value	AOR (95% CI)	*p*-Value	AOR (95% CI)	*p*-Value	AOR (95% CI)	*p*-Value	AOR (95% CI)	*p*-Value
**Gender**										
Males ^®^										
Females	1.26 (0.92–1.71)	ns	0.94 (0.68–1.30)	ns	1.05 (0.77–1.43)	ns	0.58 (0.43–1.80)	0.001	0.93 (0.68–1.28)	ns
**Age**										
15–19 ^®^										
<15 and >19	0.66 (0.48–0.91)	0.012	0.54 (0.38–0.77)	0.001	0.70 (0.50–0.97)	0.034	0.65 (0.47–0.91)	0.011	0.58 (0.41–0.80)	0.001
**Geographic background**										
Italian parents ^®^										
Foreign parents	0.92 (0.67–1.25)	ns	0.58 (0.42–0.82)	0.002	0.51 (0.37–0.70)	0.001	0.98 (0.72–1.35)	ns	0.54 (0.40–0.75)	0.001

AOR = adjusted odds ratio; CI = confidence interval; statistical analysis: multiple logistic regression; ns = not significant.

**Table 7:** Adjusted odds ratio (AOR) and 95% confidence intervals (CIs) from logistic regression analysis showing the association of lifestyle and dietary habits with gender, age, and parents’ geographic background.

	Daily Energy Drinks Consumption		Daily Soft Drinks Consumption		Fast Food at Least Once a Week		Alcohol Consumption		Daily Screen Time > 3 h		Physical Inactivity	
	AOR (95% CI)	*p*-Value	AOR (95% CI)	*p*-Value	AOR (95% CI)	*p*-Value	AOR (95% CI)	*p*-Value	AOR (95% CI)	*p*-Value	AOR (95% CI)	*p*-Value
**Gender**												
Males ^®^												
Females	0.77 (0.39–1.55)	ns	0.82 (0.56–1.22)	ns	0.85 (0.60–1.21)	ns	1.01 (0.74–1.38)	ns	1.42 (0.87–2.32)	ns	1.62 (1.02–2.59)	0.043
**Age**												
15–19 ^®^												
<15 and >19	0.73 (0.35–1.54)	ns	0.71 (0.46–1.08)	ns	0.73 (0.50–1.05)	ns	0.47 (0.34–0.66)	0.001	0.59 (0.37–0.94)	0.027	0.80 (0.47–1.34)	ns
**Geographic background**												
Italian parents ^®^												
Foreign parents	1.94 (1.00–3.76)	ns	0.78 (0.53–1.16)	ns	1.05 (0.75–1.49)	ns	0.59 (0.43–0.80)	0.001	1.45 (0.88–2.38)	ns	1.00 (0.62–1.61)	ns

AOR = adjusted odds ratio; CI = confidence interval; statistical analysis: multiple logistic regression; ns = not significant.

## Data Availability

The archived data and all the elaboration and analysis generated and used for the presentation of results in this study are fully available on request from the corresponding author.

## References

[1] United Nations Population Fund World Population Dashboard. https://www.unfpa.org/data/world-population-dashboard.

[2] Patton G.C., Sawyer S.M., Santelli J.S., Ross D.A., Afifi R., Allen N.B., Arora M., Azzopardi P., Baldwin W., Bonell C. (2016). Our future: A Lancet commission on adolescent health and wellbeing. Lancet.

[3] van Sluijs E.M.F., Ekelund U., Crochemore-Silva I., Guthold R., Ha A., Lubans D., Oyeyemi A.L., Ding D., Katzmarzyk P.T. (2021). Physical activity behaviours in adolescence: Current evidence and opportunities for intervention. Lancet.

[4] Chaudhary A., Sudzina F., Mikkelsen B.E. (2020). Promoting Healthy Eating among Young People—A Review of the Evidence of the Impact of School-Based Interventions. Nutrients.

[5] Arafa A., Yasui Y., Kokubo Y., Kato Y., Matsumoto C., Teramoto M., Nosaka S. (2024). Lifestyle Behaviors of Childhood and Adolescence: Contributing Factors, Health Consequences, and Potential Interventions. Am. J. Lifestyle Med..

[6] Liberali R., Del Castanhel F., Kupek E., Assis M.A.A.D. (2021). Latent class analysis of lifestyle risk factors and association with overweight and/or obesity in children and adolescents: Systematic review. Child. Obes..

[7] Akseer N., Mehta S., Wigle J., Chera R., Brickman Z.J., Al-Gashm S., Sorichetti B., Vandermorris A., Hipgrave D.B., Schwalbe N. (2020). Non-communicable diseases among adolescents: Current status, determinants, interventions and policies. BMC Public Health.

[8] Uddin R., Lee E.-Y., Khan S.R., Tremblay M.S., Khan A. (2019). Clustering of lifestyle risk factors for non-communicable diseases in 304,779 adolescents from 89 countries: A global perspective. Prev. Med..

[9] Veronese N., Notarnicola M., Cisternino A.M., Inguaggiato R., Guerra V., Reddavide R., Rotolo O., Zinzi I., Leandro G., Tutino V. (2020). Trends in adherence to the Mediterranean diet in South Italy: A cross sectional study. Nutr. Metab. Cardiovasc. Dis..

[10] Sinai T., Axelrod R., Shimony T., Boaz M., Kaufman-Shriqui V. (2021). Dietary Patterns among Adolescents Are Associated with Growth, Socioeconomic Features, and Health-Related Behaviors. Foods.

[11] Liu K.S.N., Chen J.Y., Ng M.Y.C., Yeung M.H.Y., Bedford L.E., Lam C.L.K. (2021). How Does the Family Influence Adolescent Eating Habits in Terms of Knowledge, Attitudes and Practices? A Global Systematic Review of Qualitative Studies. Nutrients.

[12] van Ansem W.J., Schrijvers C.T., Rodenburg G., van de Mheen D. (2014). Maternal Educational Level and Children’s Healthy Eating Behaviour: Role of the Home Food Environment (Cross-Sectional Results from the INPACT Study). Int. J. Behav. Nutr. Phys. Act..

[13] Nardone P., Pierannunzio D., Ciardullo S., Lazzeri G., Cappello N., Spinelli A., 2018 HBSC-Italia Group (2020). Dietary habits among Italian adolescents and their relation to socio-demographic characteristics. Ann. Ist. Super. Sanità.

[14] Centro di Ricerca CREA—Alimenti e Nutrizione (2018). Linee Guida per Una Sana Alimentazione, Revisione 2018.

[15] Rakić J.G., Hamrik Z., Dzielska A., Felder-Puig R., Oja L., Bakalár P., Nardone P., Ciardullo S., Abdrakhmanova S., Adayeva A. (2024). A Focus on Adolescent Physical Activity, Eating Behaviours, Weight Status and Body Image in Europe, Central Asia and Canada. Health Behaviour in School-Aged Children International Report from the 2021/2022 Survey.

[16] Ajibo C., Van Griethuysen A., Visram S., Lake A.A. (2024). Consumption of energy drinks by children and young people: A systematic review examining evidence of physical effects and consumer attitudes. Public Health.

[17] Pan B., Ge L., Lai H., Wang Q., Wang Q., Zhang Q., Yin M., Li S., Tian J., Yang K. (2021). Association of soft drink and 100% fruit juice consumption with all-cause mortality, cardiovascular diseases mortality, and cancer mortality: A systematic review and dose-response meta-analysis of prospective cohort studies. Crit. Rev. Food Sci. Nutr..

[18] Ahmed S.S., Asthana A., Dwivedi S. (2024). Fast Food Consumption Pattern and its Awareness among Youth. Act. Sci. Nutr. Health.

[19] World Health Organization Protecting Children from Unhealthy Foods and Drinks. https://www.who.int/westernpacific/news/item/19-12-2016-protecting-children-from-unhealthy-foods-and-drinks.

[20] Aonso-Diego G., Krotter A., García-Pérez Á. (2024). Prevalence of energy drink consumption worldwide: A systematic review and meta-analysis. Addiction.

[21] Scalese M., Cerrai S., Biagioni S., Benedetti E., Bastiani L., Potente R., Cutilli A., Molinaro S. (2021). Trends in energy drink and combined alcohol and energy drinks consumption among Italian high school students, 2008–2019. Drug Alcohol Depend..

[22] Cofini V., Cecilia M.R., Di Giacomo D., Binkin N., Di Orio F. (2019). Energy drinks consumption in Italian adolescents: Preliminary data of social, psychological, and behavioral features. Minerva Pediatr..

[23] Brunborg G.S., Raninen J., Burdzovic Andreas J. (2022). Energy drinks and alcohol use among adolescents: A longitudinal study. Drug Alcohol Depend..

[24] Liu M., Zhao W.Q., Zhao Q.R., Wang Y., Li S.G. (2023). The impact of the peer effect on adolescent drinking behavior: Instrumental-variable evidence from China. Front. Psychiatry.

[25] Morris H., Larsen J., Catterall E., Moss A.C., Dombrowski S.U. (2020). Peer pressure and alcohol consumption in adults living in the UK: A systematic qualitative review. BMC Public Health.

[26] Ivaniushina V., Titkova V. (2021). Peer influence in adolescent drinking behavior: A meta-analysis of stochastic actor-based modeling studies. PLoS ONE.

[27] Lees B., Meredith L.R., Kirkland A.E., Bryant B.E., Squeglia L.M. (2020). Effect of alcohol use on the adolescent brain and behavior. Pharmacol. Biochem. Behav..

[28] Gardner L.A., Stockings E., Champion K.E., Mather M., Newton N.C. (2024). Alcohol initiation before age 15 predicts earlier hazardous drinking: A survival analysis of a 7-year prospective longitudinal cohort of Australian adolescents. Addiction.

[29] Chaput J.P., Willumsen J., Bull F., Chou R., Ekelund U., Firth J., Jago R., Ortega F.B., Katzmarzyk P.T. (2020). 2020 WHO guidelines on physical activity and sedentary behavior for children and adolescents aged 5–17 years: Summary of the evidence. Int. J. Behav. Nutr. Phys. Act..

[30] Bull F.C., Al-Ansari S.S., Biddle S., Borodulin K., Buman M.P., Cardon G., Carty C., Chaput J.P., Chastin S., Chou R. (2020). World Health Organization 2020 guidelines on physical activity and sedentary behavior. Br. J. Sports Med..

[31] Guthold R., Stevens G.A., Riley L.M., Bull F.C. (2020). Global trends in insufficient physical activity among adolescents: A pooled analysis of 298 population-based surveys with 1.6 million participants. Lancet Child Adolesc. Health.

[32] Intorre F., Foddai M.S., Venneria E. (2023). Migration as Cultural Phenomenon in a Globalized World: A Pilot Study on Lifestyle and Eating Behaviours of Adolescents Living in Rome. Adolescents.

[33] Intorre F., Foddai M.S., Venneria E. (2024). Comparison of Two Indexes of Adherence to Mediterranean Diet in Adolescents: A Cross-Sectional Pilot Study. Biomed. J. Sci. Technol. Res..

[34] Intorre F., Foddai M.S., Venneria E. (2022). Mediterranean Diet Adherence in Adolescents of Different Cultures and Geographical Proveniences: A Pilot Study. Adolescents.

[35] World Health Organization (1995). Physical Status: The Use of and Interpretation of Anthropometry.

[36] Serra-Majem L., Ribas L., García A., Pérez-Rodrigo C., Aranceta J. (2003). Nutrient adequacy and Mediterranean Diet in Spanish school children and adolescents. Eur. J. Clin. Nutr..

[37] Serra-Majem L., Ribas L., Ngo J., Ortega R.M., García A., Pérez-Rodrigo C., Aranceta J. (2004). Food, youth and the Mediterranean diet in Spain: Development of KIDMED, Mediterranean Diet Quality Index in children and adolescents. Public Health Nutr..

[38] Godin G. (2011). The Godin-Shephard leisure-time physical activity questionnaire. Health Fit. J. Can..

[39] Amireault S., Godin G. (2015). The Godin-Shephard Leisure-Time Physical Activity Questionnaire: Validity evidence supporting its use for classifying healthy adults into active and insufficiently active categories. Percept. Mot. Ski..

[40] Istituto Superiore di Sanità EpiCentro—Epidemiology for Public Health. https://www.epicentro.iss.it/hbsc.

[41] Davies A., Wellard-Cole L., Rangan A., Allman-Farinelli M. (2020). Validity of self-reported weight and height for BMI classification: A cross-sectional study among young adults. Nutrition.

[42] Açik M., Çağiran Y.İ. (2022). Body awareness mediates the relationship between body mass index and lipid profiles in adolescents. J. Diabetes Metab. Disord..

[43] Hosseini S.A., Padhy R.K. (2025). Body Image Distortion (Archived). 2023 Sep 4. StatPearls [Internet].

[44] Lazzeri G., Pammolli A., Pilato V., Giacchi M.V. (2011). Relationship between 8/9-yr-old school children BMI, parents’ BMI and educational level: A cross-sectional survey. Nutr. J..

[45] Ding S., Chen J., Dong B., Hu J. (2021). Association between parental socioeconomic status and offspring overweight/obesity from the China Family Panel Studies: A longitudinal survey. BMJ Open.

[46] World Health Organization (2010). Socio-Environmentally Determined Health Inequities Among Children and Adolescents.

[47] Kocaadam-Bozkurt B., Sözlü S., Macit-Çelebi M.S. (2023). Exploring the understanding of how parenting influences children’s nutritional status, physical activity, and BMI. Front. Nutr..

[48] Hoque K.E., Hoque K.F., A/P Thanabalan R. (2018). Relationships between parents’ academic backgrounds and incomes and building students’ healthy eating habits. Peer J..

[49] Giampieri F., Rosi A., Scazzina F., Frias-Toral E., Abdelkarim O., Aly M., Zambrano-Villacres R., Pons J., Vázquez-Araújo L., Sumalla Cano S. (2024). Youth Healthy Eating Index (YHEI) and diet adequacy in relation to country-specific national dietary recommendations in children and adolescents in five Mediterranean countries from the DELICIOUS Project. Nutrients.

[50] Sincovich A., Moller H., Smithers L., Brushe M., Lassi Z.S., Brinkman S.A., Gregory T. (2022). Prevalence of breakfast skipping among children and adolescents: A cross-sectional population level study. BMC Pediatr..

[51] Monzani A., Ricotti R., Caputo M., Solito A., Archero F., Bellone S., Prodam F. (2019). A systematic review of the association of skipping breakfast with weight and cardiometabolic risk factors in children and adolescents. What should we better investigate in the future?. Nutrients.

[52] Qorbani M., Kasaeian A., Rafiemanzelat A., Sheidayi A., Djalalinia S., Nouri K., Rastad H., Salimi D., Ghaderi K., Motlagh M.E. (2021). Social inequalities in meal skipping patterns among children and adolescents: The CASPIAN–V study. Obes. Sci. Pract..

[53] Ofori-Asenso R., Owen A.J., Liew D. (2019). Skipping breakfast and the risk of cardiovascular disease and death: A systematic review of prospective cohort studies in primary prevention settings. J. Cardiovasc. Dev. Dis..

[54] Palomar-Cros A., Srour B., Andreeva V.A., Fezeu L.K., Bellicha A., Kesse-Guyot E., Hercberg S., Romaguera D., Kogevinas M., Touvier M. (2023). Associations of meal timing, number of eating occasions, and night-time fasting duration with incidence of type 2 diabetes in the NutriNet-Santé cohort. Int. J. Epidemiol..

[55] Zhang Z., Tan J., Luo Q. (2024). Associations between breakfast skipping and outcomes in neuropsychiatric disorders, cognitive performance, and frailty: A Mendelian randomization study. BMC Psychiatry.

[56] Rosi A., Scazzina F., Giampieri F., Álvarez-Córdova L., Abdelkarim O., Ammar A., Aly M., Frias-Toral E., Pons J., Vázquez-Araújo L. (2025). Lifestyle factors associated with children’s and adolescents’ adherence to the Mediterranean diet living in Mediterranean countries: The DELICIOUS Project. Nutrients.

[57] Masini A., Dallolio L., Sanmarchi F., Lovecchio F., Falato M., Longobucco Y., Lanari M., Sacchetti R. (2024). Adherence to the Mediterranean Diet in children and adolescents and association with multiple outcomes: An umbrella review. Healthcare.

[58] Rutigliano I., Mansueto M.L., Canestrale R., Giorgio R., Sacco M., Pastore M.R. (2024). Children’s diet assessed with the Mediterranean Diet Index: The finding of new eating habits and their impact on a cohort of Italian children. Ann. Dell’Ist. Super. Sanità.

[59] Iaccarino Idelson P., Scalfi L., Valerio G. (2017). Adherence to the Mediterranean diet in children and adolescents: A systematic review. Nutr. Metab. Cardiovasc. Dis..

[60] Stanaway J.D., Afshin A., Ashbaugh C., Bisignano C., Brauer M., Ferrara G., Garcia V., Haile D., Hay S.I., He J. (2022). Health effects associated with vegetable consumption: A Burden of Proof study. Nat. Med..

[61] Afshin A., Sur P.J., Fay K.A., Cornaby L., Ferrara G., Salama J.S., Mullany E.C., Abate K.H., Abbafati C., Abebe Z. (2019). Health effects of dietary risks in 195 countries, 1990–2013; 2017: A systematic analysis for the Global Burden of Disease Study 2017. Lancet.

[62] FAO (2021). Fruit and vegetables—Your dietary essentials. The International Year of Fruits and Vegetables Background Paper.

[63] Geraldo R., Santos C.S., Pinto E., Vasconcelos M.W. (2022). Widening the perspectives for legume consumption: The case of bioactive non-nutrients. Front. Plant Sci..

[64] Zargarzadeh N., Mousavi S.M., Santos H.O., Aune D., Hasani-Ranjbar S., Larijani B., Esmaillzadeh A. (2023). Legume consumption and risk of all-cause and cause-specific mortality: A systematic review and dose-response meta-analysis of prospective studies. Adv. Nutr..

[65] Marventano S., Izquierdo Pulido M., Sánchez-González C., Godos J., Speciani A., Galvano F., Grosso G. (2017). Legume consumption and CVD risk: A systematic review and meta-analysis. Public Health Nutr..

[66] Kim J.M., Lee E. (2021). Association between soft-drink intake and obesity, depression, and subjective health status of male and female adults. Int. J. Environ. Res. Public Health.

[67] Hu H., Song J., MacGregor G.A., He F.J. (2023). Consumption of soft drinks and overweight and obesity among adolescents in 107 countries and regions. JAMA Netw. Open.

[68] Jia P., Luo M., Li Y., Zheng J.S., Xiao Q., Luo J. (2021). Fast-food restaurant, unhealthy eating, and childhood obesity: A systematic review and meta-analysis. Obes. Rev..

[69] Jiao J., Moudon A.V., Kim S.Y., Hurvitz P.M., Drewnowski A. (2015). Health implications of adults’ eating at and living near fast food or quick-service restaurants. Nutr. Diabetes.

[70] Protano C., Valeriani F., De Giorgi A., Angelillo S., Bargellini A., Bianco A., Bianco L., Caggiano G., Colucci M.E., Coniglio M.A. (2023). Consumption of energy drinks among Italian university students: A cross-sectional multicenter study. Eur. J. Nutr..

[71] Scuri S., Petrelli F., Tesauro M., Carrozzo F., Kracmarova L., Grappasonni I. (2018). Energy drink consumption: A survey in high school students and associated psychological effects. J. Prev. Med. Hyg..

[72] Scafato E., Ghirini S., Gandin C., Matone A., Manno V., Vichi M., CSDA Working Group (2024). Rapporto ISTISAN 24/3 “Epidemiologia e Monitoraggio Alcol-Correlato in Italia e Nelle Regioni. Valutazione Dell’Osservatorio Nazionale Alcol Sull’impatto del Consumo di Alcol ai Fini Dell’implementazione delle Attività del Piano Nazionale Alcol e Salute e del Piano Nazionale di Prevenzione.”.

[73] Wilhite K., Booker B., Huang B.H., Antczak D., Corbett L., Parker P., Noetel M., Rissel C., Lonsdale C., Del Pozo Cruz B. (2023). Combinations of physical activity, sedentary behavior, and sleep duration and their associations with physical, psychological, and educational outcomes in children and adolescents: A systematic review. Am. J. Epidemiol..

[74] World Health Organization (2024). Physical Activity Measurement and Surveillance in Youth: Report of a Scoping and Planning Meeting.

[75] Devi K.A., Singh S.K. (2023). The hazards of excessive screen time: Impacts on physical health, mental health, and overall well-being. J. Educ. Health Promot..

[76] Kandola A., Lewis G., Osborn D.P.J., Stubbs B., Hayes J.F. (2020). Depressive symptoms and objectively measured physical activity and sedentary behavior throughout adolescence: A prospective cohort study. Lancet Psychiatry.

[77] Zablotsky B., Arockiaraj B., Haile G., Ng A.E. (2024). Daily screen time among teenagers: United States, July 2021–December 2023. NCHS Data Brief.

[78] Ricardo L.I.C., Wendt A., Costa C.D.S., Mielke G.I., Brazo-Sayavera J., Khan A., Kolbe-Alexander T.L., Crochemore-Silva I. (2022). Gender inequalities in physical activity among adolescents from 64 Global South countries. J. Sport Health Sci..

[79] Rosselli M., Ermini E., Tosi B., Boddi M., Stefani L., Toncelli L., Modesti P.A. (2020). Gender differences in barriers to physical activity among adolescents. Nutr. Metab. Cardiovasc. Dis..

[80] Strain T., Wijndaele K., Garcia L., Cowan M., Guthold R., Brage S., Bull F.C. (2020). Levels of domain-specific physical activity at work, in the household, for travel and for leisure among 327,789 adults from 104 countries. Br. J. Sports Med..

